# UK quality statements on end of life care in dementia: a systematic review of research evidence

**DOI:** 10.1186/s12904-015-0047-6

**Published:** 2015-10-19

**Authors:** Bridget Candy, Margaret Elliott, Kirsten Moore, Victoria Vickerstaff, Elizabeth L Sampson, Louise Jones

**Affiliations:** Marie Curie Palliative Care Research Department, UCL Division of Psychiatry, 6th Floor, Wing B, Maple House, 149 Tottenham Court Road, London, W1T 7NF UK

**Keywords:** Dementia, Palliative care, Policy, Review

## Abstract

**Background:**

Globally, the number of people who die with dementia is increasing. The importance of a palliative approach in the care of people with dementia is recognised and there are national polices to enhance current care. In the UK implementation of these polices is promoted by the National Institute for Health and Care Excellence (NICE) Dementia Quality Standards (QS). Since publication of the QS new care interventions have been developed.

**Aim:**

To explore critically the current international research evidence on effect available to inform NICE Dementia QS relevant to end of life (EOL) care.

**Design:**

We used systematic review methods to seek the research evidence for three statements within the Dementia QS. These are those that recommend: (1) a case management approach, (2) discussing and consideration of making a statement about future care (SFC) and (3) a palliative care assessment (PCA). We included evaluative studies of relevant interventions that used a comparative design, such as trials and cohort studies, and measured EOL care outcomes for persons dying with moderate to severe dementia. Our primary outcome of interest was whether the intervention led to a measurable impact on wellbeing for the person with dementia and their family. We assessed included studies for quality using a scale by Higginson and colleagues (2002) for assessment of quality of studies in palliative care, and two authors undertook key review processes. Data sources included Cinahl, Embase, and PsychINFO from 2001 to August 2014. Our search strategy included free text and medical subject headings relevant to population and recommended care.

**Results:**

We found seven studies evaluating a care intervention; four assessed SFC, three PCA. None assessed case management. Studies were of weak design; all used retrospective data and relied on others for precise record keeping and for accurate recall of events. There was limited overlap in outcome measurements. Overall reported benefits were mixed.

**Conclusions:**

Quality statements relevant to EOL care are useful to advance practice however they have a limited evidence base. High quality empirical work is needed to establish that the recommendations in these statements are best practice.

## Background

Dementia is a progressive neurodegenerative disease for which there is currently no disease modifying treatment. Across the world a large and increasing number of people are living with dementia. In the UK approximately 835,000 people have dementia [1] and estimates suggest that of those aged 65 years and over, one in three will die either with or from the disease [2]. The need for appropriate care and support for people with dementia is a recognised priority [3].

The importance of a palliative approach in the care of people with dementia is recognised at international level [4, 5], and there are national initiatives that aim to guide clinicians, and policy-makers to provide the most appropriate care at the end of life (EOL) [6]. The UK Department of Health’s *End of Life Care Strategy* is an example [7]. It is a general strategy irrespective of disease since many principles of care are common for all who are approaching death. However, compared with cancer or other chronic diseases, people with dementia may have different EOL needs, including communication and cognitive difficulties [8, 9]. There are policy initiatives specific to dementia including, in the UK, *Living Well with Dementia: a national dementia strategy* [10], these though do not provide principles for EOL care.

Care quality indicators are used (for example in the UK, the Netherlands, Australia) to help drive improvement in areas of high priority [11]. They are measurable aspects of care for which quality standards (QS) are developed [12]. In the UK, they are developed by the National Institute for Health and Care Excellence (NICE) and there are two QS specific to dementia: QS13, published in 2014 is on living well with dementia [13]; and QS1, published in 2010 is on health and social care which has three statements relevant to EOL care [14]. There is no agreed definition of QS, in general they are based on clinical guidelines and/or published evidence and are composed of concise statements that aim to set guidance on what is minimally acceptable as good practice. In the recent political climate they have assumed a significant profile as central, although not mandatory, in their application to delivery of the NHS. (12) A recent systematic review of the international literature on clinical guidelines in dementia highlighted the limited number of recommendations provided in guidelines on EOL care [15].

However, since the millennium, there have been interventions introduced that are relevant to EOL for people with dementia, including advance care planning (ACP), a palliative approach to care and case management. Advance care planning (ACP) involves discussion of future care preferences to develop an understanding of an individual‘s wishes should they become unable to make decisions for themselves. This may lead to the individual making a statement about future care (SFC) [16]. This has particular resonance in dementia where people may become unable to participate in decisions about their care in the later stages of dementia or at the EOL. The benefits of this intervention though are not fully established and evaluations on ACP continue to be undertaken [17]. There is also international work that aims to clarify both the nature of a palliative approach to care for people with dementia and how it might be implemented [5]. For example, the *Palliative Excellence in Alzheimer Care Efforts (PEACE) programme* is a disease management program that incorporates a palliative care focus from the diagnosis of dementia through its terminal stages [18]. Case management involves a pro-active approach to care, identifying the needs of people with complex conditions and developing a personalised care plan [19]. It often involves establishing a key worker to co-ordinate care and this approach is relevant at all stages of dementia. We are aware of evaluations of such interventions in dementia, although they may not all involve care at the EOL [20–25].

Given that the UK Department of Health depends upon QS to drive improvements in care, it is important that such initiatives are informed by research evidence [26]. A review of this evidence is valuable to inform future thinking on both recommendations for care and priorities for new research.

## Aim

In this paper, we explore critically the current international research evidence on effect available to inform the following NICE Dementia QS1 statements relevant to EOL care:

- Statement 4 (S4): People with dementia have an assessment and an ongoing personalised care plan agreed across health and social care that identifies a named care coordinator and addresses their individual needs.
- Statement 5 (S5): People with dementia, while they have capacity, have the opportunity to discuss and make decisions, together with their carer/s, about the use of advance statements, advance decisions to refuse treatment, lasting power of attorney and preferred priorities of care.
- Statement 9 (S9): People in the later stages of dementia are assessed by primary care teams to identify and plan their palliative care needs.

Our primary outcomes of interest were wellbeing of the person with dementia and of their family.

## Method

We used systematic review methods guided by the Cochrane Handbook [27] and the Preferred Reporting Items for Systematic Reviews and Meta-Analyses guidelines [28].

### Inclusion criteria

We sought first to identify randomised controlled trials (RCTs) however we were aware that the number of such studies might be limited. This is in part due to the ethical issues involved in withholding treatments or care from people with dementia and/or those nearing the EOL. Involvement in research may be perceived as burdensome and upsetting for patients and their families [29]. We therefore also included more pragmatic evaluative studies that used any comparative design such as quasi-controlled trials, observational and cohort studies with a comparative arm and before-and-after studies.

We included studies of populations with dementia or documented cognitive deficit. We did not require specific diagnostic inclusion criteria for dementia, as many people never receive a formal diagnosis [30]. We set two criteria of increased relevance for EOL: (1) participants at the EOL were described as having moderate, severe or advanced dementia, (2) study assessed outcomes related to EOL care, such as pain control, place of death or type of care (e.g. use of palliation or life-sustaining treatments). For S5 we set an additional criterion that the studies involved the process of considering preparing SFC at a period when the person with dementia still had capacity.

The criteria for the interventions selected were that they should combine all of the core requirements of each individual statement, which the research team interpreted as:

- S4: (1) a named care coordinator, (2) an assessment of health and social care needs, (3) a personalised care plan, and (4) integration/co-ordination of care services.
- S5: (1) people with early dementia who retained mental capacity and had made decisions regarding future healthcare preferences including the use of advance statements and/or advance decisions to refuse treatment and/or lasting power of attorney and/or preferred priorities of care.
- S9: (1) an assessment of palliative care needs, (2) a palliative care plan, and (3) delivery within a primary care or community setting.

The setting of the delivery of the intervention could be the participant’s home, a care or nursing home (NH) or other community facility.

### Exclusion criteria

We excluded studies if they were concerned only with acute care delivered within secondary care settings. We did not include studies of people at the EOL with mild dementia. If the severity of dementia was mixed, we excluded the study unless more than 33 % of the sample had moderate or severe dementia and where possible we reported these results only.

### Information sources

We searched five citation databases, CINAHL, Embase, PsychINFO, Cochrane and Web of Science, from January 2001 to August 2014. We chose this start date because from 2001 there has been increased recognition by governments and other public agencies, and therefore a likely increase in research in dementia.

### Search strategy

The search strategy was developed through consultation within our specialist dementia research group and refined using test searches with medical subject headings (MESH) and text terms. The search terms for each statement are shown in Table 1. Abstracts of citations identified were screened; S4 and S9 by ME, S5 by ME and KM. A subsample of each search was independently checked by BC to ensure consistency when applying inclusion criteria. Full text of citations which appeared relevant were checked for eligibility by ME and BC. Reference lists were checked for further relevant studies.

**Table 1:** Search terms for three NICE Quality Care Statements

Search terms for dementia: Dementia or Alzheimer^a^ or lewy body or lewy bodies or cognitive impair^a^ or capacity impair^a^ or lack^a^ capacity or memory loss	
*Search terms used for standard S4*:	
Key worker or key carer or named worker or named care co-ordinator or named carer or community psychiatric nurse or liaison worker or link worker or community health nursing.	
*Search terms used for standard S5:*	
	(Advance^a^ (plan or care or directive or decision or contract or statement)) or living will^a^ or right to die or power of attorney or ulysses (contract^a^ or directive^a^) or PPC or preferred priorities of care or anticipatory care plan.
*Search terms used for standard S9*:	
(Primary care or general practice or community care or care in the community or family physician or community nurse or practice nurse) AND (Palliative care or end-of-life or symptomatic medicine or end of life or supportive care).	

^a^plurals or other word endings

### Assessment of quality and risk of bias

We assessed the quality of studies using an approach developed by Higginson et al. [31] that is relevant for reviews in palliative care. The approach grades by study design and key aspects of the methodological process such as analysis and outcome measurement. The approach is detailed in Table 2.

**Table 2:** Definitions used for the grades of evidence

*Grade I: Randomised controlled trial (RCT)*	
IA Calculation of sample size and accurate, standard definition of outcome variables	
IB Accurate and standard definition of outcome variables.	
IC Neither of the above	
*Grade II: Prospective study with a comparison group (non randomised controlled trial, observational study) or retrospective study which controls for confounding variables*	
IIA Calculation of sample size^a^ and accurate, standard definition of outcome variables and adjustments for the effects of important confounding variables	
IIB One or more of the above	
IIC Neither of the above	
*Grade III Retrospective or observational or cross-sectional studies*	
IIIA Comparison group, calculation of sample size and accurate, standard definition of outcome variables	
IIIB One or more of the above	
IIIC Neither of the above.	

^a^If sample size above 1000 or more we considered this criteria to have been reached

### Outcomes of interest

We were interested in evaluating whether the intervention led to a measurable impact on wellbeing for the person with dementia and their family, including aspects of care of relevance at the EOL. For all statements our primary outcomes were:

- At the EOL QOL, symptoms and distress. These could be captured using validated tools such as the Symptom Management-End-of-Life in Dementia [32] and the Comfort Assessment in Dying with Dementia (CAD-EOLD) [32].
- The families’ satisfaction with care. This could be captured by the Satisfaction With Care at the End- Of-Life in Dementia scale [32].
- Types of care and treatment such as whether the person was admitted to a hospice or was hydrated artificially.
- Place of death.

For each study if reported we detailed whether the intervention was actually implemented, such as documentation of a SFC.

Our secondary outcomes of interest were

- Measures of family wellbeing and QOL.
- Economic costs.

Data extraction and analysis

We extracted characteristics of the studies directly into tables. These included country of origin, study design, population characteristics, description of intervention, type of analysis and results.

We sought, if appropriate, to combine trial results in a meta-analysis. Extraction of the data was undertaken by one researcher (ME/BC) and checked by another (ME/BC/VV/KM). To allow the reader to consider the effect of the intervention we sought to present fully the findings, including the size of effect. We sought to standardise the presentation of results. For analysis using continuous variables to compare outcomes between participants receiving the intervention and not receiving the intervention we present, if appropriate, the mean difference (MD) and standard deviation (SD). For those using dichotomous data we present, if appropriate, the odds ratio (OR) and 95 % confidence interval (CI).

## Results

**Fig. 1:**
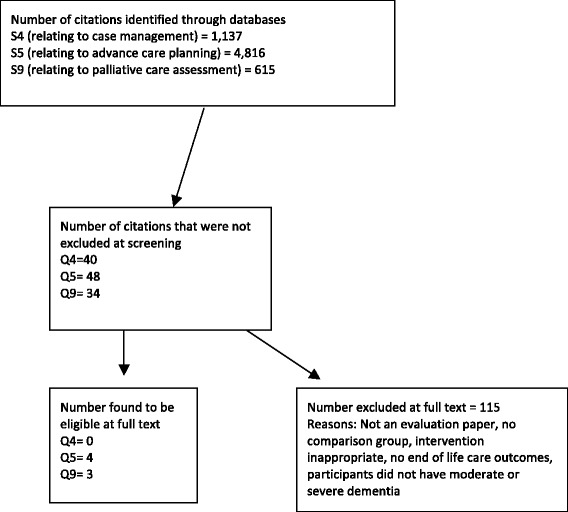
Flow chart of review process

The databases searched identified 5,548 citations. At screening for eligibility, for 122 it was necessary to ascertain eligibility by reading full text articles. Of these, agreement was easily reached to exclude 104 as they did not report an evaluation or the intervention was ineligible. A further 10 studies were judged by consensus of the wider research team as not eligible [18, 20–25, 33–35]; they are listed with reasons for exclusion in Table 3. The remaining seven studies were eligible [36–42]. A flowchart describing the screening processes is shown in Fig. 1.

**Table 3:** List of excluded studies with reasons for exclusions

Study	Reason for exclusion
Bass et al. 2003 [33]	Intervention ineligible
Callahan et al. 2006 [20]	Not evaluating QOL in patients with advanced dementia
Challis et al. 2002 [23]	No outcome on QOL at the EOL
Eloniemi-Sulkava et al. 2001 [24]	No outcome on QOL at the EOL
Eloniemi-Sulkava et al. 2009 [25]	No outcome on QOL at the EOL
Fortinsky et al. 2009 [21]	No outcome on QOL at the EOL
Samus et al. 2014 [34]	No outcome on QOL at the EOL
Shega et al. 2008 [18]	Whilst appropriate outcomes were compared with those that received hospice care with those that didn’t both groups were assessed for palliative care needs. (QS5)
Specht et al. 2009 [35]	No outcome on QOL at the EOL
Vickrey et al. 2006 [22]	No outcome on QOL at the EOL

These are the studies excluded where because of lack of clarity it was necessary to discuss their inclusion in the review at a regular research meeting

Three included studies were from USA [37, 39, 40], two from Belgium [41, 42], one from Canada [36] and one from the UK [38]. None were RCTs. All reported data collected retrospectively. They either (1) compared one group of persons with dementia who underwent the intervention with another group who did not [39–42] or (2) compared data collected before and after implementation of the intervention in the same NH site [36–38]. Combining any of the studies in a meta-analysis was not appropriate because of study design and heterogeneity across studies. No studies were rated as the highest grade of quality. Four were graded as IIA as they were cohort studies with comparative groups, had large samples, controlled in their analyses for known key confounders such as age and had robust outcome measures [39–42]. The other three were graded as IIIB as they were before-and-after studies and used robust outcome measures [36–38].

We found no studies that assessed outcomes specifically relevant to EOL care that may have supported S4 which recommended a case management approach. Of note we excluded the *PEACE programme* [18], which describes case management in dementia with a palliative care focus. We excluded this as the absence of a comparison group made interpretation of the EOL care outcomes difficult. However, we acknowledge that benefits of care co-ordination prior to the terminal phase are reported [43].

Four of the seven eligible studies were of an intervention on SFC as recommended for S5 [39–42] and three for S9; which involved a palliative care assessment [36–38].

### Statement 5 (S5): People with dementia, while they have capacity, have the opportunity to discuss and make decisions, together with their carer/s, about the use of advance statements, advance decisions to refuse treatment, Lasting Power of Attorney and Preferred Priorities of Care

Four studies were relevant to S5 [39–42]. All were post-mortem studies comparing EOL outcomes dependent on whether or not prior to loss of mental capacity the deceased person had made a documented SFC. All studies provided descriptions of a SFC including (1) an expressed desire to limit care [39], (2) discussions about the goals and desired direction of care, particularly EOL care, in the event that the individual is or becomes incompetent to make decisions [41, 42], or (3) documentation of individual wishes with respect to life-sustaining treatment described as a ‘living will’ or their choice of a surrogate decision maker, as in a durable power of attorney for healthcare [40].

EOL care outcomes included the type of care provided, symptoms and distress experienced, and place of death. All studies used regression analysis to control for potential confounders. Three of the studies involved at the EOL participants with severe dementia [39, 41, 42]. The fourth included participants with cognitive deficits [40]. Table 3 provides details on the studies.

Two studies were undertaken by the same team but used different samples [41, 42]. All participants were resident in a NH. They both used cross-sectional data on deaths from large nationally representative surveys of NH residents. In one survey, data were collected on 764 residents within 345 NH [41], and in the other on 101 residents in 69 NH [42]. Data on care outcomes were collected by questionnaire.

In the other two studies their samples examined participant data from the Health and Retirement Study (HRS), a large nationally representative longitudinal cohort study of people aged 60 years or over. The study data were linked to Medicare claims spending in the last six months of life and measures of aggressive or life-sustaining treatment, such as feeding tube placement, intensive care use, and in-hospital death. Both report data on just under 4,000 individuals. Proxy respondents at post-mortem interview were asked if there was a SFC. One study details that proxy respondents were most commonly (48 %) an adult child [39].

We graded the quality of these four studies at IIA. Overall the studies showed mixed impact of the intervention.

#### Statement about future care

The proportion of participants at EOL who had a documented SFC varied. In both studies by Vandervoort and colleagues a SFC was reported as present for 17 % of the participants [41, 42]. In one study, 1 % (n = 8) of participants had appointed a legal representative [42], and the most frequent treatment directives reported were do-not-hospitalise (DNH) (2.5 %) and do-not-resuscitate (DNR) (2.1 %) orders.

In the evaluations using data from the HRS study, one reports that for 44.9 % (of 3746) who had loss of capacity (due to any diagnosis) a SFC was in place [39], and in the other a SFC was in place for 36.5 % of the sample with severe dementia [40].

#### Quality of life, symptoms and distress in the person with dementia

Two of the studies explored QOL, symptoms and distress; one used the CAD-EOLD on comfort assessment [42], the other the Edmonton Symptom Assessment Scale or nurse report. [41] One study found no statistical differences in the last week of life between those who had a SFC and those who did not in terms of symptoms (See Table 4) [41]. In the other study regression models showed no statistically significant difference in overall scores on comfort between those with and without a SFC [42]. However, for three of the twelve subscale analyses there was a significant reduction in emotional distress. This was for

**Table 4:** Studies addressing issues in Statement 5: Study characteristics, intervention content and outcomes reported

Study & country	Design	Population	Intervention content, analysis^a^ and number with a statement about future care (SFC) or related document	Results: Impact on care outcomes of having or not having advanced directives or related documents^a^		
Nicholas 2014 et al. [39] USA	Retrospective study which controls for confounding variables Data from 3876 participants of the Health & Retirement Study (HRS) who died between 1998 and 2007. Study exit interview (after death) by knowledgeable informant (KI)	Age 65 years and over, consented to share Medicare claims data for last 6 months of life. Includes nursing home (NH) (n = 1812) and community dwelling people (n = 2064). 21.7 % severe dementia, 43.1 % mild dementia or cognitive impairment, 35.2 % normal cognition (as assessed at last HRS interview –mean 436 days before death – using validated cognition measures). Results only reported for severe dementia.	Comparison of end-of-life care with those that had and those that didn’t have a written advance directive (AD) or “living will” as stated by proxy respondent in post-mortem interview. General liner model and logistic regression to generate predicted spending and probabilities of use in last six months of life. Adjustments were made for patient characteristics, including cognitive functioning, an AD and their interaction, stratified by NH use in last 6 months. 36.4 % of people with severe dementia had a treatment limiting AD (40 % of NH, 27.4 % of community dwelling).	REGRESSION: results presented for subgroup with severe dementia only		
				Medicare spending ($1,000)		
					Community-dwelling	NH
				No SFC	32.2	24.7
				SCF	20.7***	22.5
				Hospital death (%)		
					Community-dwelling	NH
				No SFC	31.8	20.6
				SFC	13.9***	14.6**
				Life sustaining treatment (%)		
					Community-dwelling	NH
				No SFC	19.8	11.6
				SFC	10.6	9.8
				ICU use (%)		
					Community-dwelling	NH
				No SFC	19.6	10.8
				SFC	10.2***	7.1
				Difference in results of AD and no AD not significant except where **/*** *P* value ** < 0.05, ***p < 0.01		
Silveira 2010 et al. [40] USA	Retrospective study which controlled for confounding variables. Data from 3746 participants in HRS Study who died between 2000 and 2006. Study exit interview (after death) by knowledgeable informant (KI) – a relative most commonly as adult child	Age 60 years and over, died 2000–2006, 46 % had cognitive impairment prior to death (but not dementia specific) as stated by KI	Comparison of end-of-life care as stated by KI in post-mortem interview with those that had and those that didn’t have a written SFC or Power of Attorney (PoA).	REGRESSION: Adjusted odds ratio and (95 % confidence interval) for presence versus absence of a living will or a PoA^a^		
					SFC	PoA
			Multiple logistic regression. Adjustments made for socio-demographic and clinical characteristics. 42.5 % required decision-making at end of life, 70 % of whom lacked capacity, 67.6 % of whom had SFC. 83 % who requested limited care and 97 % who requested comfort care received care consistent with preferences	Hospital death	0.71 (0.47, 1.07)	0.72 (0.55, 0.93)^a^
				All care	0.33 (0.19, 0.56)^a^	0.54 (0.34, 0.86)^a^
				Limited Care	1.79 (1.28, 2.50)^a^	1.18 (0.75, 1.85)
				Comfort Care	2.59 (1.06, 6.31)^a^	2.01 (0.89, 4.52)
				All care is care under any circumstances to prolong life, Limited care is care in certain circumstances, this is as opposed to all care possible in order to prolong life, Comfort care is comfortable and pain-free while forgoing extensive measures to prolong life.		
				^a^Difference statistically significant		
Vandervoort 2012 et al. [41] Belgium	Retrospective study which controls for confounding variables	NH resident.	Presence of a written doctors orders to withhold treatment, such as do-not-hospitalise or do-not-resuscitate, or advance patient directive (SFC), such as a “living will” as stated by nurse in post-mortem questionnaire. This questionnaire also captured care outcomes, Comparison of outcomes dependent on presence of SFC. Multivariate logistic regression model. Outcomes only on SFC presented. (The others are not relevant to this review).	REGRESSION: Adjusted odds ratio and 95 % confidence interval presented on SFC and patient directives only		
	Study of deaths in NH of residents with dementia. All NH (594) invited to participate. Post death questionnaire completed by nurse.	Diagnosis of dementia, and severity, as stated by nurse.		Symptoms in the last week of life using Edmonton Symptom Assessment Scale		
		Died in 2-month study period in 2006. 72 % female, 78 % 80 years and above. 63 % severe and 37 % moderate dementia. 764 deaths in 345 NH (58 % response rate).		Pain		0.72 (0.37, 1.40)
				Tiredness		1.08 (0.54,2.15)
				Nausea		0.55 (0.20,1.51)
				Depression		1.42 (0.73,2.77)
				Anxiety		1.38 (0.73,2.58)
				Drowsiness		0.78 (0.40,1.51)
				Appetite		1.14 (0.55,2.40)
				Shortness of breath		1.19 (0.63, 2.23)
				QOL last week of life		1.14 (0.58,2.24)
				Mildness of death		1.70 (0.70, 4.12)
				Death in hospital or palliative care unit		2.09 (0.92, 4.72)
				All odds ratios not significant		
Vandervoort 2014 et al. [42] Belgium	Retrospective study which controls for confounding variables. Study of NH of residents with dementia who died within a 3-month period in 2010 (representative sample using random cluster-sampling). Post death questionnaire completed by nurse, GP, family member or friend & NH administrator.	Diagnosis of dementia as stated by nurse or GP. 58 % female, 84 % 80 years and above. 51 % very severe dementia, 25 % severe dementia, 24 % moderate or mild dementia	Comparison of scores on the Comfort Assessment in Dying in Dementia Scale (CAD-EOLD).	REGRESSION: Mean (standard deviation) score in total and subscales of the CAD-EOLD^a^		
		Relative available to complete questionnaire. 101 deaths in 69 NH (58 % response rate).	Multivariate logistic regression adjusting for age, gender, level of dementia and sentinel events.		SFC	No SFC
			17.5 % had a written advanced directive, 56.7 % had GP orders	Total	31.9 (7.1)	29.1 (6.3)
				Physical distress	8.8 (2.9)	8.2 (2.2)
				Dying symptoms	8.6 (2.4)	8.0 (2.6)
				Emotional distress	10.2 (2.3)**	9.0 (2.3)
				Wellbeing	6.2 (1.9)	5.9 (1.9)
					DNH	No DNH
				Total	32.8 (6.4)	29.1 (6.4)
				Physical distress	8.8 (2.9)	8.2 (2.2)
				Dying symptoms	9.1 (2.2)	8.0 (2.6)
				Emotional distress	10.4 (1.8)***	9.0 (2.4)
				Wellbeing	6.4 (2.0)	5.9 (1.9)
					DNR	No DNR
				Total	32.9 (5.6)	29.1 (6.5)
				Physical distress	9.3 (2.7)	8.1 (2.7)
				Dying symptoms	8.6 (2.3)	8.1 (2.7)
				Emotional distress	10.6 (1.7)****	9.0 (2.4)
				Wellbeing	6.5 (1.6)	5.9 (1.9)
					PDMA	No PDMA
				Total	29.1 (7.6)	29.4 (6.3)
				Physical symptoms	8.2 (2.6)	8.1 (2.3)
				Dying symptoms	7.8 (3.0)	8.0 (2.6)
				Emotional distress	9.4 (3.3)	9.1 (2.3)
				Wellbeing	5.6 (0.9)	5.9 (1.9)
				^a^All adjusted odds ratios reported as not significant apart from: ** 2.99 (CI 1.1, 8.3) ***2.54 (0.8, 7,7), ****3.45 (CI 1.1,11).		

^a^All trials presented the outcomes reported here as a primary outcome. Results presented are the most complete reported in the published paper. *CAD-EOLD* = Comfort Assessment in Dying in Dementia Scale, *DNH* = do-not-hospitalise, *DNR* = do not resuscitate, *KI* = knowledgeable informant, *GP* = general practitioner/primary care physician, *HRS* = Health & Retirement Study, *NH* = nursing home, *PDMA* = proxy decision maker assigned, *PoA* = Power of Attorney. *QOL* = quality of life, *SFC* = statement about future care

- Having a SFC compared to not having a SFC (OR 2.99; CI 1.1, 8.3),
- The SFC requested DNH (OR 2.54; CI 0.8, 7.7),
- The SFC requested DNR (OR 3.45; CI 1.1, 11).

#### Types of care or treatment

Two studies explored if having a SFC made a difference to whether the person with dementia received aggressive or life-sustaining treatment [39, 40]. One explored outcomes in two samples: those with dementia living in the community outside of a NH and those residing in a NH [39]. They found no significant difference in the proportion receiving aggressive or life-sustaining treatment between those who had a SFC and those who did not, but those who lived in the community (outside of a NH) and had a SFC were significantly less likely to be admitted to an intensive care unit (10.2 % v 19.6 % *p* value <0.01).

The other study found, in people with loss of capacity due to any diagnosis, that those who had a SFC were significantly less likely to receive aggressive or life-sustaining treatment (OR 0.33; CI 0.19, 0.56) [40]. Likewise they found that those who had appointed a durable power of attorney for healthcare were less likely to receive such treatment (OR 0.54; CI 0.34, 0.86). They also explored whether there was a greater chance that those with a SFC compared to those who did not would receive ‘comfort care’ (described as being comfortable and pain-free while forgoing extensive measures to prolong life) and limited care only (described as limited care in certain situations, as opposed to all care possible in order to prolong life). For both limited and comfort care analysis showed an association with having a SFC (OR 1.79 CI 1.28, 2.50; OR CI 2.59, CI 1.06, 6.31 respectively).

#### Place of death and other outcomes

Three studies looked at place of death [39–41]. One found those with a SFC compared to those without were significantly less likely to die in a hospital; in the community sample 13.9 % versus 31.8 % and in the NH 14.6 % versus 20.6 % respectively [39]. In another study there was no difference in regards to a SFC (OR 0.71; CI 0.47, 1.07) but there was a difference if a durable power of attorney for healthcare had been made (OR 0.72; CI 0.55, 0.93) [40]. The third study found no difference, however it differed its analyses, it compared whether a person died in hospital or a palliative care unit with whether or not they died in a NH [41]. One study reported economic costs and found the presence of a SFC reduced Medicare spending in community dwelling persons [39]. No studies reported family outcomes, adverse effects or economic costs.

### Statement 9 (S9): People in the later stages of dementia are assessed by primary care teams to identify and plan their palliative care needs

Table 5 provides details on study characteristics and their results.

**Table 5:** Studies addressing issues discuss in Statement 9: Study characteristics, intervention content and outcomes reported

Study and country	Design	Population	Intervention content, data collection, analysis and number with palliative care assessment	Results^a^: Impact on care outcomes of assessment in the community to identify, and plan palliative care needs				
Arcand et al. 2009 [36] Canada	Pre and post intervention study.	Relatives of residents who died with advanced dementia, and where dementia was the main physician recorded diagnosis. pre intervention Mean age 91 years and 90 % female and post intervention. 87 years and 71 % female.	Intervention educational Delivered by geriatric nurse specialist for all care home staff and physicians. Topics included symptom control & palliative care in advanced dementia, advance care planning & medical guidelines for considering prognosis. Information booklet for use by staff and to give to family members.	Mean difference in scores and confidence intervals pre and post intervention^a^				
	One 387 bed nursing home (NH).	48 deceased residents, 27 pre-intervention, and 21 post-intervention.	Data collection 4–16 months post bereavement. Family satisfaction with care using validated scale “After death bereaved family member interview/ NH version”.	Communication		1.40 [−3.04, 0.24]		
	Unit of analysis NH		Analysis t-tests statistics	Care according to				
				Patient’s wishes		−0.10 [−1.18, 0.98]		
				Symptom control		−0.80 [−1.96, 0.36]		
				Dying with dignity		0.10 [−1.22, 1.42]		
				Family emotional				
				Support		1.00 [−2.85, 0.85]		
				Satisfaction		−1.00 [−2.05, 0.05]		
				^a^using “After death bereaved family member interview/ Nursing Home version”.				
				None of the mean differences were signficiant.				
Hanson et al. 2005 [37] US	Pre and post intervention study, 7 NH with another 2 NH acting as a control. This was to gauge temporal trends.	All residents, 43 % had dementia diagnosis and 76 % cognitive impairment.	Intervention educational Plan-do-study-act design. NH identified staff members to form interdisciplinary palliative care team who attended one day conference; education on hospice enrolment and services, pain management, advance care plans and communication. Then monthly in house education and support (x6 sessions) available to all clinical staff; help with designing procedures & protocols and the use of assessment tools.	Results in numbers:				
						Pre (%)		Post (%)
						*N* = 345		*n* = 346
	Unit of analysis NH	Mean age 82 years, 74 % white, 81 % female.	Data collection from medical records and after death interview at least three months after the death.	Hospice care		4.0		6.8*
		345 residents pre intervention and 346 post intervention	Analysis chi-squared statistics	Pain assessed		18		60*
		113 residents in 2 control NHs No significant differences between intervention and control NHs at baseline		Receiving pain				
				Medication		77		81
				Receiving non-drug				
				Treatment for pain		15		34*
				DNR order		58		65*
				DNR flagged in chart		45		60*
				Documented discussion on preferences		4		17*
				*Statistically significant, *P* value equal or less than 0.05, chi-squared test				
Livingston, 2013 et al. [38] UK	Pre and post intervention study,	Residents with dementia of any severity (most advanced dementia MMSE mean = 5)	Educational interventional 10 session training program for all staff covering structured listening, empathy communication skills, advance care planning and preferred place of care.		Pre	Post	X^2^	*P* value
	One 120 bed NH.	Residents with dementia of any severity (most advanced dementia MMSE mean = 5)	Data collection from medical records & after-death bereaved family member interview	EOL talk	04/30	13/28	15.2	0.001
	Unit of an analysis the NH	59 deaths, 30 1 year pre-study, 29 1 year post study.	Analysis before and after intervention using t-tests and Mann Whitney for means or medians according to data distribution	DNR orders	04/28	16/22	17.4	0.001
				EOL talk +				
				DNR order	01/04	12/16	5.3	0.06
				Deaths in care home	14/30	22/29	5.3	0.02
				Intervention				
				In line with wishes	05/07	13/13	4.1	0.04
				Days in hospital				
				Last 3 months	4	1.25	29.0^a^	0.22
				Care				
				satisfaction	7.5 (1.3)	9.1 (2.4)	17.6^a^	0.06
				^a^ *t* test or Mann–Whitney				

^a^Results presented as the most complete reported in the published paper, *EOL* = end of life, *DNR* = Do not resuscitate, *NH* = nursing home, *X* ^2^ = Chi –Squared test

Three studies were relevant to S9 [36–38]. They all involved residents of NH who had died with dementia and in each the focus of the intervention involved assessment and planning of palliative needs. All were before-and-after studies where the unit of analysis was the NH. One study was from the USA [37], one from Canada [36], and one the UK [38].

All were staff educational intervention programs designed to facilitate recognition of the needs of residents with dementia who were approaching the EOL. Topics included symptom assessment and care. The largest study included 458 residents within nine NH; seven homes took part in the intervention and two acted as controls to gauge temporal trends in care [37]. The other two studies involved one NH [36, 38]. In one study all participants had at EOL advanced dementia [36], in another the majority did [38] and in the other the majority had cognitive deficits [37].

The studies were graded at IIIB. Overall the evidence on the impact of the palliative care intervention was mixed.

#### Person with dementia quality of life, symptoms and distress

One study reported on symptoms of pain or distress at the EOL [36], it found no significant mean change in pain symptom scores or dying with dignity.

#### Types of care and treatment

One study found a significant increase in hospice referral (before 4 % of residents were referred whereas following the intervention 6 % were referred, *p* value > 0.05) [37]. There were also reported statistically significant changes evident in four of the five other outcomes reported on aspects of care:

- Assessed for pain (pre intervention 18 %, and post 60 %),
- Received non-pharmacological treatment (pre 15 %, post 34 %),
- Had a DNR order (pre 58 %, post 65 %),
- Had a DNR indicator or ‘flag’ in a chart outside of their notes (pre 45 %, post 60 %).

There was no significant difference in the number receiving pain medication.

#### Satisfaction with care at the end of life

Two studies reported satisfaction with care at the EOL [36, 38]. They both used the validated After Death Bereaved Family Member Interview Toolkit, neither reported significant change in family satisfaction with care.

#### Place of death and other outcomes.

One study reported the place of death; it found a significant increase in deaths occurring within the NH as opposed to hospital, from 47 % (14/30) pre-intervention to 76 % (22/29) post (*p* value 0.02) [38]. In addition, those whose wishes had been recorded were more likely to die in their preferred place of care.

No studies reported on family outcomes, adverse events or economic costs.

## Discussion

We sought to identify and examine the quality and quantity of evidence underpinning the UK quality statements designed to help drive improvements in care for the growing number of people dying with dementia. We located evidence to inform the three statements of the NICE Quality Standards (QS1) that are most pertinent to EOL care of people with moderate to severe dementia (S4, S5, S9). We found no studies that met our inclusion criteria that related specifically to S4, which recommends a case management approach. Whilst for this statement we identified nine studies that matched our criteria on interventions (see Table 3), we were not able to include them in this review due to the nature of their study design and outcome data. In particular, a lack of a comparison group made interpretation of the EOL care outcomes difficult. Other excluded studies were trials of case management but they did not assess the benefit for participants with advanced dementia at the EOL. The case management approach highlights aspects of care that we would assume are relevant to a person irrespective of the stage of their disease, in particular a named care coordinator responsible for assessment and an ongoing personalised care plan. Whilst it may be reasonable to expect that a case management approach initiated earlier in the illness might be continued and have benefits right up until the time of death, stronger evidence for the effectiveness of case management at this particular time would be helpful.

For the other statements the evidence was limited by the number and heterogeneity of the included studies. We identified four studies relevant to S5, which recommends that early in dementia discussion is undertaken about making a statement about future care (SFC). Two of these studies explored the impact of making advanced decisions on QOL, symptoms and distress at the EOL. In both no significant differences in these domains were found between those who had made a SFC and those who had not [41, 42]. However, in one study a weaker (subgroup) analysis showed evidence to suggest that the presence of a SFC may reduce emotional distress in the person with dementia [42]. There was mixed evidence on the effect of a SFC on types of treatment at the EOL [39, 40], for instance in one study there was a significantly lower chance of receiving aggressive or life sustaining treatment if the person had a SFC [40], but this was not found in the other study [39]. We also found mixed results on whether the person died in hospital, with one study finding an association between not having a SFC and dying in a hospital [39], and another study reported no significant difference [41]. The differences in findings between the studies could be due to heterogeneity such as measuring impact using different scales or because of different characteristics in the populations or settings.

Three studies were relevant to S9 which recommends palliative care assessment [36–38]. Outcomes reported across these studies varied and so it is difficult to draw conclusions. For example, following the intervention in one study there was no significant improvement in symptom control [35], in another study there was significantly more assessment of pain but no increase in the number of participants receiving pain medication [36]. In one study, post intervention significantly more people with dementia received hospice care [37] and in another more received care at the EOL in line with their wishes [38]. The differences in findings between the studies could be due to heterogeneity such as how the outcome is measured, in the population considered and differences or local limitations in delivery of the intervention. Moreover our findings suggest for all statements there is a need for further research to clarify the impact of what is recommended. Our results do not suggest that these quality statements should be withdrawn.

## Strengths and limitations

This review was challenged by the quality of the evidence identified. Although most of the included studies involved large samples there were limitations with study design. All used retrospective data and relied on others for precise record-keeping and for accurate recall of events. Three were before-and-after studies, with limited use of an external comparison group. Therefore it is difficult to establish cause and effect. Changes reported may have occurred even if the interventions had not been implemented. For example, NH staff may, because of increased recognition in the public domain of palliative care, have started to assess the EOL needs of residents.

None of the studies were specifically designed to explore the benefits of interventions recommended in the UK quality statements. Moreover, we had difficulties in clearly defining our population of interest at the EOL. People with dementia may die *from* dementia when it is advanced when they are likely to have lost mental capacity, or they may die *with* dementia as a co-morbid condition when it is mild. Palliative and EOL needs as understood by the quality standards are relevant in both scenarios. However for the reason of increased relevance we considered only evidence from studies of those facing the EOL either with *moderate to severe* dementia or who had documented loss of mental capacity.

Our review was limited by the numbers of eligible studies and research design. In addition, we focused only on UK statements. However, although there are guidelines on dementia in other countries that are specific to EOL, we are unaware of quality statements elsewhere that aim to drive forward aspects of practice across different healthcare settings. The evidence is also limited in its application; three of the studies were conducted in the USA where healthcare provision differs from that available in other countries including the UK. Only one study was undertaken in the UK. It is also difficult to comment on the overall applicability of the findings as there was limited evidence of consistency. Few measurements overlapped in studies and there was considerable variation in the reported effectiveness of the interventions. This variation may be explained by contextual differences, but it should be remembered that the design of these studies is weak, increasing the risk of bias in their results.

We searched widely using large citation databases and developed our search terms, refining iteratively with test searches and consultation within the research team that comprised of experts in palliative care, dementia and primary care. We used a number of terms for each statement (S4, 5, 9) that illustrated its main aims. Whilst we think that we were inclusive and broad in our approach, the terminology within each statement lacked specificity and may have been subject to our interpretation; a different group of researchers may have captured different studies. However, the evidence available was so scarce that we think it is unlikely that we have overlooked any major research in this field.

To our knowledge no other published systematic review has been undertaken to evaluate the evidence relevant to the NICE QS1 on EOL care. There is a recent Cochrane review on case management for people with dementia in one care setting, the person’s home [44]. It found evidence to support the benefit of this intervention. Our review differed in that it only critiqued studies involving an intervention at EOL. At this stage many people with dementia are residing in a care home. Moreover, our focus involved a QS recommending three aspects of care that may be described in case management programs; (i) an assessment and an ongoing personalised care plan agreed across health and social care (ii) that identifies a named care coordinator and (iii) addresses their individual needs. Not all case management approaches may include all three.

Other related research includes a systematic review of advance care planning in general populations which it also revealed a lack of high quality studies [17]. It may not be surprising that the evidence is limited. We acknowledge the potential difficulties in undertaking research in populations at the EOL and also in people with dementia, in particular issues of capacity and consent of participants, obtaining agreement from personal or professional consultees and the ethics of randomising to a control group. In addition there may be difficulties in engaging care staff because of high staff turnover in NH and the perception that research will take-up too much of staff time.

The limitations in the research evidence relevant to other high profile healthcare clinical guidelines and initiatives are recognised, including those of the World Health Organization [45]. Moreover, where there is a need to provide guidance despite limited evidence, it is rational to draw upon evidence derived from elsewhere, such as extrapolation of findings from other populations, from expert clinical opinion and from patient and public consultation [46].

## Conclusions

### Clinical and methodological implications

Despite the weaknesses found in the literature, we do not suggest that clinicians and commissioners should no longer consult quality standards. These standards are useful to advance practice. Instead we suggest that, without robust supporting evidence, it is important to bear in mind that what is recommended may have the potential for harmful effects or, more plausibly, there is a risk that precious funds and resources are not being used to best effect [47].

As the numbers dying with dementia increases worldwide more high quality evidence to inform approaches to EOL care is needed. Detection of real intervention effects requires adequate control of any bias that may distort the effect so as to ensure that there is no false assumption of effectiveness and lack of possible harm. [48] We suggest that rigorous prospective evaluation of current and new approaches is possible and desirable with use of consent and assent procedures guided by the Mental Capacity Act 2005. Key issues include the use of appropriate comparator groups either in controlled observational studies or in RCT perhaps using cluster designs and multi-level approaches to data analysis. Where trials cannot be conducted, data from matched sites collected prospectively can be analysed using difference-in-difference methods that take account of policy changes over time [49]. Approaches to measuring quality of care for those with significant cognitive impairment should be informed by careful selection of outcome measures that can be accurately rated either through use of proxies or by direct observation. Assessment of QOL in those with impaired capacity is an area that requires further investigation [50]. Such measures can be supported by process data, selected and collected with similar care. Whilst international evidence is of value, it is important to take account of the context of the local healthcare systems when planning innovations and to gain societal views by undertaking patient and public consultation. Likewise whilst we have looked at the evidence that could be used to underpin the NICE QS in regards to dementia EOL care, if QS continue to be given high priority by the UK government there is a case for independent evaluation of their use and impact [12].

## Abbreviations

ACP: Advanced care planning
AD: Advance directive
CAD-EOLD: the Comfort Assessment in Dying with Dementia
CI: Confidence interval
DNH: Do not hospitalise
DNR: Do not resuscitate
EOL: End of life
HRS: Health & Retirement Study
KI: Knowledgeable informant
MD: Mean difference
MESH: Medical subject headings
NICE: National Institute for Health and Care Excellence
NH: Nursing home
OR: Odds ratio
PCA: Palliative care assessment
QOL: Quality of life
QS: Quality standard
RCT: Randomised controlled trial
SD: Standard deviation
SFC: Statement about future care
